# Supplementary material for: Fungal Pathogens Associated with Strawberry Crown Rot Disease in China
Source: J Fungi (Basel). 2022 Nov 2;8(11):1161. doi: 10.3390/jof8111161 (PMC9698672; doi:10.3390/jof8111161)
Supplement: Supplementary file 1 [file jof-08-01161-s001.zip › jof-1940083-supplementary.pdf]

Table S1. The GenBank accession number of test strain for identification and classification of strawberry crown rot pathogens.

Species	Isolate	GenBank Accession Number					
		ITS	ACT	CAL	CHS	GAPD	EF-1α
C. fructicola	JD-ZJ-3-2	MW513775	MW513825	MW513829	MW513833	MW513837	
	JD-ZJ-5	MW513776	MW513826	MW513830	MW513834	MW513838	
	JD-ZJ-9	MW513777	MW513827	MW513831	MW513835	MW513839	
	JD-ZJ-12	MW513778	MW513828	MW513832	MW513836	MW513840	
C. siamense	ZS-HX-54	MW513762	MW513852	MW513900	MW513876	MW513924	
	ZS-HX-64	MW513763	MW513853	MW513901	MW513877	MW513925	
	ZS-HX-77	MW513764	MW513854	MW513902	MW513878	MW513926	
	ZS-HX-85	MW513765	MW513855	MW513903	MW513879	MW513927	
	ZS-HX-111	MW513766	MW513856	MW513904	MW513880	MW513928	
	ZS-ZJ-6	MW513767	MW513857	MW513905	MW513881	MW513929	
	ZS-ZJ-20	MW513768	MW513858	MW513906	MW513882	MW513930	
	ZS-ZJ-32	MW513769	MW513859	MW513907	MW513883	MW513931	
	ZS-ZJ-49	MW513770	MW513860	MW513908	MW513884	MW513932	
	ZS-ZJ-53	MW513771	MW513861	MW513909	MW513885	MW513933	
	JD-HX-A-3	MW513751	MW513841	MW513889	MW513865	MW513913	
	JD-HX-A-6	MW513752	MW513842	MW513890	MW513866	MW513914	
	JD-HX-A-12	MW513753	MW513843	MW513891	MW513867	MW513915	
	JD-HX-A-16	MW513754	MW513844	MW513892	MW513868	MW513916	
	JD-HX-A-21	MW513755	MW513845	MW513893	MW513869	MW513917	
	JD-ZJ-16	MW513761	MW513851	MW513899	MW513870	MW513923	
	JD-TXZ-2	MW513756	MW513846	MW513894	MW513871	MW513918	
	JD-TXZ-7	MW513757	MW513847	MW513895	MW513872	MW513919	
	JD-TXZ-12	MW513758	MW513848	MW513896	MW513873	MW513920	
	JD-TXZ-15	MW513759	MW513849	MW513897	MW513874	MW513921	
	JD-TXZ-18	MW513760	MW513850	MW513898	MW513875	MW513922	
	YH-YX-1	MW513772	MW513862	MW513910	MW513886	MW513934	
	YH-YX-6	MW513773	MW513863	MW513911	MW513887	MW513935	
	YH-YX-10	MW513774	MW513864	MW513912	MW513888	MW513936	
F. commune	ZS-ZJ-46-2	MW513789					MW513819
	ZS-HX-91	MW513790					MW513820
	ZS-HX-107	MW513791					MW513821
	JD-HX-A-27-2	MW513792					MW513822
F. equiseti	JD-HX-A-19-2	MW513793					MW513823
F. oxysporum	ZS-ZJ-9-3	MW513785					MW513814
	ZS-ZJ-38-2	MW513786					MW513815
	JD-HX-A-10	MW513779					MW513808
	JD-HX-A-11	MW513780					MW513809
	JD-HX-A-13-3	MW513782					MW513811
	JD-HX-A-29	MW513783					MW513812
	JD-HX-B-1	MW513781					MW513810
	JD-HY-15	MW513784					MW513813
	YH-HX-4	MW513788					MW513817
	YH-HX-16	MW513787					MW513816
F. solani	ZS-ZJ-40-2	MW513794					MW513818
F. tricinctum	ZS-ZJ-33-2	MW513795					MW513824
Clonostachys rosea	ZS-ZJ-46-1	MW513804					
	ZS-ZJ-51-3	MW513803					
Stemphylium lycopersici	ZS-HX-72	MW513801					
	ZS-HX-96-2	MW513802					
Curvularia trifolii	ZS-HX-60-2	MW513805					
Epicoccum sorghinum	ZS-HX-55-3	MW513798					
	ZS-HX-80	MW513796					

	ZS-HX-84	MW513797	
Phoma herbarum	ZS-ZJ-24-1	MW513799	MW513806
	ZS-ZJ-53-4	MW513800	MW513807
